# LncRNA T-UCR Uc.339/miR-339/SLC7A11 Axis Regulates the Metastasis of Ferroptosis-Induced Lung Adenocarcinoma

**DOI:** 10.7150/jca.65017

**Published:** 2022-03-28

**Authors:** Ning Zhang, Jian Huang, Mei Xu, Yuli Wang

**Affiliations:** 1 Division of Gastroenterology, Ganzhou People's Hospital, the Affiliated Ganzhou Hospital of Nanchang University, Ganzhou, Jiangxi 341000, China; 2 Department of Hematology, Wenzhou Central Hospital, Theorem Clinical College, Wenzhou Medical University, Wenzhou, Zhejiang 325000, China.; 3 Department of Oncology, Ganzhou People's Hospital, the Affiliated Ganzhou Hospital of Nanchang University, Ganzhou, Jiangxi 341000, China.

**Keywords:** Uc.339, LncRNA T-UCR, miR-339, lung adenocarcinoma, ferroptosis

## Abstract

Lung adenocarcinoma progression is closely linked to ferroptosis suppression. Emerging studies have found that the expression of its related gene SLC7A11 may be regulated by LncRNA. However, the mechanism of LncRNA in affecting the development of SLC7A11-mediated lung adenocarcinoma remains unclear. Here, we identified a Uc.339/miR-339/SLC7A11 axis that involves LncRNA T-UCR Uc.339-mediated repression of miR-339 and affects the expression of SLC7A11 to participate in tumor metastasis and development. In this study, we identified Uc.339 as upregulated in patients with lung adenocarcinoma. RAP-qPCR proved that LncRNA Uc.339 competitively binds to pri-miR-339 and inhibits the production of mature miR-339. The interaction between miR-339 and SCL7A11 was confirmed by luciferase reporter assay. The Uc.339/miR-339/SLC7A11 axis regulated the proliferation, migration and invasion of A549 and H1299cells *in vitro* by affecting ferroptosis. Finally, in mouse xenograft models, knocking down Uc.339 in LLC cells was able to inhibits tumor growth by blocking the axis of Uc.339/miR-339/SLC7A11 i*n vivo*, but miR-339 inhibitors could reverse this inhibition. Taken together, our results uncovered a Uc.339/miR-339/SLC7A11 axis that leads to defects in the ferroptosis in lung cancer, and constitutes a potential mechanism that drives the metastasis of lung adenocarcinoma.

## Introduction

Lung cancer is the most common malignant tumor globally with high morbidity and mortality^1^. NSCLC is the main type of lung cancer, which accounts for 80-85%^2^-^4^. Moreover, lung adenocarcinoma has been reported to be the most predominant subtype of NSCLC that is experiencing the fastest growth in incidence^5^. Compared with the traditional chemoradiotherapy, targeted therapy significantly improved the long-term survival rate of lung adenocarcinoma patients^6^. Despite improved short-term survival rates, long-term survival rates dismal.

LncRNA T-UCR is a new type of lncRNA discovered in recent years. They are frequently located at fragile sites and other cancer-associated genomic regions and are dysregulated in several types of solid and hematological malignancies, compared to their normal tissue counterpart^7^. Changes in the expression of T-UCRS and its relationship with tumors have been found in a variety of cancers. Sana's group identified Uc.73A and Uc.338 as oncogens in colorectal cancer (CRC) and hepatocellular carcinoma (HCC)^8^, ^9^; Uc.8 was able to promote bladder carcinogenesis by interacting with miR-596^10^. Recently, it was shown that transcribed Uc.339 is overexpressed in HCC cells, contributing to a pro-tumoral HCC microenvironment^11^. Another study found that Uc.339 is also abnormally upregulated in lung cancer^8^, ^12^. Collectively, these findings support a role for T-UCRs in human carcinogenesis. However, much about the mechanism and consequences of dysregulation of T-UCR in human cancers remains unknown.

The mechanism by which lncRNA T-UCR functions is as an adsorption sponge for primary microRNA. Through the transcribed ultra-conservative region, lncRNA T-UCR can compete with Drosha-DGCR8 to combine with specific primary miRNA (pri-miRNA) and regulate its shearing to reduce the expression level of miRNA^13^, ^14^. MiR-339 has been negatively associated with tumor occurrence, development and metastasis and appears to act as a tumor suppressor^15^, ^16^. Previous studies demonstrated that transcribed uc.339 is upregulated in archival NSCLC samples, functioning as a decoy RNA for miR-339-3p, -663b-3p, and -95-5p^12^. With that, we guessed that Uc.339 may reduce the expression of miR-339 through adsorption and binding, thereby weakening the effect of miR-339 in inhibiting related carcinogenic targets.

Ferroptosis is an iron-dependent, non-apoptotic form of cell death characterized by the accumulation of reactive oxygen species in cells^17^, ^18^, which is related to the occurrence and development of a variety of tumors^19^-^22^. It also constitutes one of the mechanisms of the occurrence and development of lung adenocarcinoma^23^, ^24^. Several recent studies reported that SLC7A11 was closely negatively correlated with ferroptosis and the suppression of SLC7A11 induced ferroptosis^25^. The *in vitro* experiments showed the high SCL7A11 expression level increase the proliferation, invasion, and migration ability of lung cancer^26^. Therefore, we speculated that SLC7A11 may exert a carcinogenic effect in lung adenocarcinoma by inhibiting the death of tumor cells.

In this study, we proved that the combined regulation relationship of Uc.399/miR-339 is an important link that affects the occurrence and development of lung adenocarcinoma. At the same time, a Uc.339/miR-339/SLC7A11 axis was also discovered, which is composed of Uc.339-mediated miR-339 inhibition and SLC7A11 up-regulation. It inhibits ferroptosis in A549 and H1299 cells to promote tumor proliferation and metastasis.

## Materials and methods

### Cell culture and Reagents

Lung cancer cells (A549, H1299, 16HBE and PC-9) were obtained from the Type Culture Collection of the Chinese Academy of Sciences (Shanghai, China). The cells were cultured in DMEM with 10% fetal bovine serum (Gibco) and 100 U/mL penicillin/streptomycin (Invitrogen Corporation, Carlsbad, CA, USA) at 37 °C incubator (Sanyo, Osaka, Japan) with 5% CO_2_.

MiR-339 mimics, negative control mimics, miR-339 inhibitors, and negative control inhibitors were all purchased from Exiqon (Woburn, MA, USA). Uc.339 knockdown shRNA plasmid (Uc.339 KD) was purchased from Santa Cruz Biotechnology (Santa Cruz, CA, USA). Uc.339 pcDNA3.1 (+) plasmid (Uc.339 OE) was purchased from Addgene (Cambridge, MA, USA).

### Western Blot Analysis

Total protein extracts and Western blot (WB) analysis were performed as in previous studies^27^. Primary antibodies dilutions were performed as the following: SCL7A11 (#12691, Cell Signaling), Beta-actin (a5441, Sigma-Aldrich). All antibodies were used at 1 mg/mL working concentration in TBST with 5% BSA. The membrane was further probed with horseradish peroxidase (HRP)-conjugated rabbit antimouse immunoglobulin G (IgG) (Santa Cruz Biotechnology, 1:2,000), and the protein bands were visualized using enhanced chemiluminescence (Amersham Pharmacia, Piscataway, NJ, USA).

### qRT-PCR

Total RNA was extracted using TRIzol (Invitrogen) according to the manufacturer's protocol. cDNA was generated using SuperScript™ IV VILO™ Master Mix (Invitrogen). Uc.339, miR-339, SCL7A11 mRNA was normalized to GAPDH mRNA. The primer sequences were as follows:

Uc.339-F, 5'-TCTTGGACCAACAAGTAGCC-3';

Uc.339-R, 5'-GTCGGGATCCGTCATCTTG-3';

miR-339-F, 5'-TGATGACATCAAGAAGGTGGTGAAG-3';

miR-339-R, 5'-TCCTTGGAGGCCATGTGGGCCAT-3';

SLC7A11-F, 5'-TCTTGGACCAACAAGTAGCC-3';

SLC7A11-R, 5'-GTCGGGATCCGTCATCTTG-3';

PTGS2-F, 5'-GGACACTGAGCAAGAGAGGC-3';

PTGS2-R, 5'-TTATGGGGGTCTGGGATGGA-3';

NOX1-F, 5'-TTACAGGCTGCTGCTCAGTC-3';

NOX1-R, 5'-AGGCGTCAAAGTAAGCCCAG-3';

COX2-F, 5'-CAGCAGCAGCAGACTCAAGA-3';

COX2-R, 5'-GCACAGGCTGCTTCTGAAAG-3';

ACSL4-F, 5'-AGTTTACAGGCTGCTGCTCA-3';

ACSL4-R, 5'-CAGGCGTCAAAGTAAGCCCA-3';

FTH1-F, 5'-TAGGCTTGTGGCCAAGACCTTCAT-3';

FTH1-R: 5'-CGGTCCTGGCCCTGACAAA-3';

GPX4-F, 5'-CCTTCATCTTTAGCCGATCCA-3';

GPX4-R, 5'-GACTCCAGGGAAGTTGAGAAA-3';

GAPDH-F, 5'-ATCAATGGAAATCCCATCACCA-3';

GAPDH-R, 5'-GACTCCACGACATACTCAGCG-3';

The results were calculated using the 2^-△△CT^ method, and the results are expressed as the mean ± SD^28^.

### RAP-PCR and RAP-Western Blot

Synthesize a specific biotinylated probe that hybridizes with Uc.339. Take 1 × 10^7^ cells to be tested, wash them with PBS and dissolve them in 500 uL cell lysis buffer, and then sonicate them to release the particles. Add specific biotinylated probe and antisense probe, and incubate at room temperature for 2h. Then add 50ul blocking streptavidin magnetic beads (Invitrogen) and incubate at 4°C for 4h. Magnetically separate the magnetic beads from the cell debris, and then wash them three times on the magnetic rack with cold lysis buffer and cold PBS. Use the Trizol method to extract the target RNA, and then perform Realtime PCR for verification.

### Proliferation Assays

Proliferation assays was performed as previously described^29^.

### Wound-healing Assay

The wound-healing assay was performed as previously described^30^.

### Transwell

Transwell were performed as previously described^31^.

### Luciferase Activity Assay

The wild-type and mutated miR-339 putative targets on IL-6 3′ UTR were cloned into pGL3-promoter vector. The cells (2 × 10^4^) were co-transfected with 500 ng pGL3-IL-6-wild-type (WT) and pGL3-IL-6-mutant (Mut) constructs with miR-339 mimics. Each sample was co-transfected with 50 ng pRL-SV40 plasmid expressing renilla luciferase to monitor the transfection efficiency. A luciferase activity assay was performed 48h after transfection with the dual luciferase reporter assay system (Promega, WI, USA). The relative luciferase activity was normalized to the renilla luciferase activity.

### GSH Analysis

GSH was detected with the GSH detection kit in accordance with the manufacturer's instructions. Briefly, A549 and H1299 cells were seeded into 6-well cell culture plates with 2 × 10^5^ cells per well and cultured continuously for 12 h. Different intervention reagents were added to the cultural plates and cultured continuously for 48 h. Cells were harvested and washed twice with PBS. The cells were resuspended in reagent I and frozen and thawed three times with liquid nitrogen. A 20 μL supernatant from the cell suspension centrifuged at 8000 rpm was mixed with 140 μL of reagent II and 40 μL of reagent III and detected with a multifunctional enzyme marker (Varioskan Flash, Thermo Scientific) at the wavelength of 412 nm.

### Intracellular ROS Detection

The intracellular ROS generation was detected using a ROS assay kit (Abcam) according to the manufacturer's instructions. Briefly, A549 and H1299 cells were seeded into 6-well cell culture plates with 2 × 10^5^ cells per well and cultured continuously for 12 h. Different intervention reagents were added to the cultural plates and cultured continuously for 48 h. Tumor single cell suspensions were incubated with DCFH‐DA probe at 1:1000 dilution in serum‐free RPMI medium at 37°C for 30 min, then washed with serum‐free RPMI medium three times and detected by a fluorescent reader (TECAN) with excitation at 488 nm.

### Animal experiences

#### Xenograft model

1.0 × 10^5^ Uc.339-NC and Uc.339-KD LLC cells, with or without miR-339 inhibitors in 150 μL of 0.9% normal saline were injected subcutaneously into 6-8 weeks old C57BL/6J mice (n=5 mice per group). The mice were sacrificed after 4 weeks, and the tumors were collected. Each tumor sample was snap-frozen in liquid nitrogen or fixed immediately in 4% paraformaldehyde overnight at 4 °C.

#### Experimental lung metastatic model

6-8 weeks mice were divided into 4 groups randomly. mice were injected with 2.5 × 10^5^ wild type LLC cells, Uc.339 OE-LLC cells, with or without miR-339 inhibitors, respectively, through the lateral tail vain. The mice were killed after 4 weeks by carbon dioxide asphyxiation followed by cervical dislocation to ensure death. The lungs were removed, rinsed with PBS, and the number of metastatic foci on the lung surface was counted. The pulmonary lobes were subsequently kept in 4% paraformaldehyde for later paraffin embedding and hematoxylin and eosin staining.

All animal studies were conducted in accordance with the NIH Guidelines for the Care and Use of Laboratory Animals.

### Statistical Analyses

All experiments were performed at least three times, and the results are presented as the mean ± standard deviation. Significance was calculated by two-tailed *t-test* for two group comparisons, while ANOVA test with Bonferroni correction was used for multiple group comparisons. The cases from TCGA analysis consisted of Lung adenocarcinoma (LUAD) samples with overall survival information and Uc.339 expression computed. The patient clinical information for the TCGA patients with LAUD from cbioportal (https://www.cbioportal.org). Survival curves were plotted using the Kaplan-Meier method and compared using the log-rank test. Correlations were determined by Pearson's correlation.

## Results

### Uc.339 was upregulated in lung cancer, and promoted the proliferation and metastasis of lung adenocarcinoma cells

**Figure 1:**
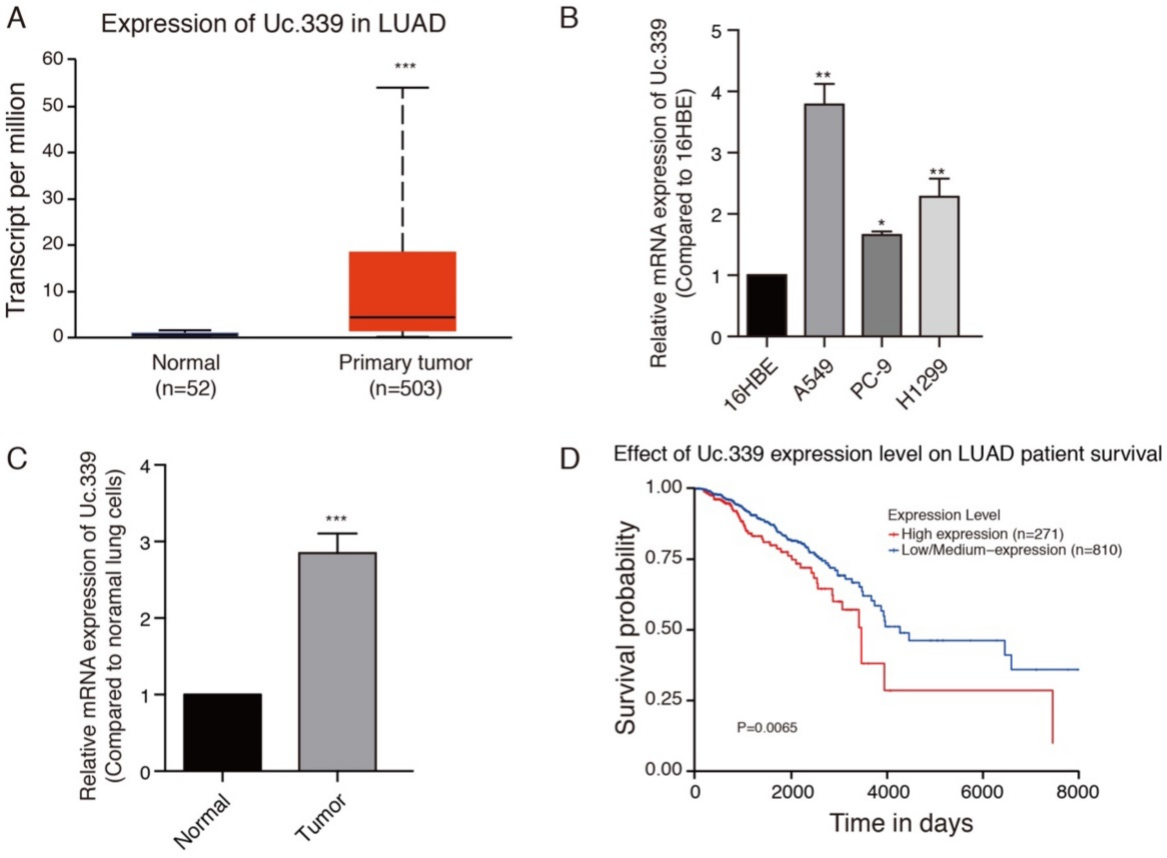
** Uc.339 was upregulated in lung cancer and lung adenocarcinoma cells. a** Histogram represent Uc.339 expression levels in lung adenocarcinoma tissues (n=503) and normal gastric tissues (n=52) according to the data from TCGA database. **b** qRT-PCR analysis of Uc.339 expression in human bronchial epithelial cells (16HBE) and lung adenocarcinoma cells (A549, PC-9 and H1299). **c** qRT-PCR analysis of Uc.339 expression in cells extracted from healthy lung tissues and tumor tissues of mice. **d** Kaplan-Meier analysis of overall survival in patients with variable Uc.339 expression according to the data from the TCGA database (*P*=0.0065). Each bar represents the mean ± SD of three independent experiments; *P<0.05; **P<0.01; ***P<0.001.

**Figure 2:**
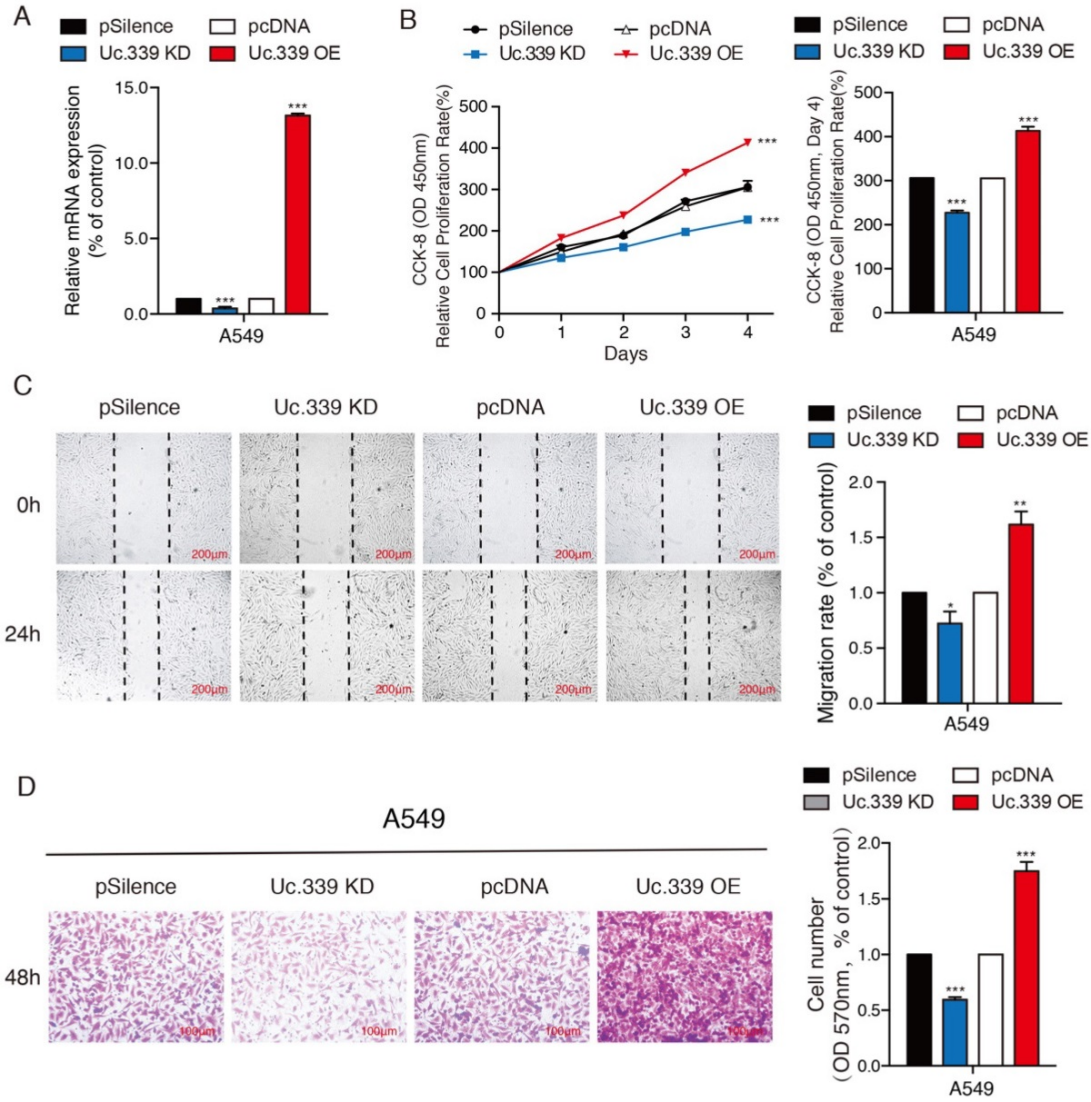
** Uc.339 promoted the proliferation and metastasis of lung adenocarcinoma cells. a** qRT-PCR analysis of Uc.339 expression in the A549 cells with Uc.339 knockdown or Overexpression. **b** CCK-8 assay showing the proliferation rate of A549 cells with Uc.339 knockdown or overexpression. **c** Wound-healing assay showing the migration ability of A549 with Uc.339 Knockdown or overexpression. Scale bar, 200μm. **d** Transwell assay showing the invasion ability of A549 cells with Uc.339 Knockdown or overexpression. Scale bar, 100μm. Each bar represents the mean ± SD of three independent experiments; *P<0.05; **P<0.01; ***P<0.001.

Increased expression of Uc.339 has been described in NSCLC^12^. We asked whether such an upregulation also occurred in LUAD primary samples. To investigate the role of Uc.339 in the development of lung cancer, the gene expression date of 555 lung cancer were downloaded from the Cancer Genome Atlas (TCGA) database. Uc.339 expression was first analyzed in 503 lung cancer tissues and 52 normal lung tissues, and we found that Uc.339 was significantly upregulated in lung cancer tissues (Fig. 1A). As showed in Fig. 1B, the expression of Uc.339 in lung adenocarcinoma cells (A549, PC-9 and H1299) were higher than that in human bronchial epithelial cells (16HBE). In order to further prove this conclusion *in vivo*, we extracted primary cells from healthy lung tissues of mice and primary cells from lung tumor tissues of experimental lung metastasis model mice, and tested their UC.339 expressions respectively. Consistent with the results of *in vitro* cell experiments, the expression of Uc.339 in cells extracted from tumor tissues was significantly increased compared with cells extracted from healthy tissues (Fig. 1C). Kaplan-Meier survival curve analysis showed that the overall survival of the patients with high levels of Uc.339 expression was obviously shorter than that of patients with low levels of Uc.339 expression (Fig. 1D). Therefore, we speculated that Uc.339 may be closely related to the development of lung adenocarcinoma. To verify this guess, we constructed A549 and H1299 cell lines with the plasmid of lncRNA Uc.339 stable over-expression. Knockdown of Uc.339 in cells was achieved by transfection with Uc.339 shRNA (Fig. 2A, Fig. S1A). We observed that in the cell proliferation experiment, the Uc.339 overexpression group showed a faster cell proliferation rate in each time period, while the Uc.339 knockdown tumor cells proliferation rate was markedly lower than that of the control group (Fig. 2B, Fig. S1B). A wound-healing assay was performed to investigate the effect of Uc.339 on the horizontal migration of tumor cells by observing the wounded confluent monolayers of each group of cells. The wound of Uc.339-knockdown cells was obviously broader than that of control cells. On the contrary, Uc.339 overexpression markedly promoted the spreading of the tumor cells along the edges of the wound compared with the behavior observed in the control cells (Fig. 2C, Fig. S1C). We conducted the Transwell to explore the effect of Uc.339 KD/OE on tumor cells invasion. Similar to the results of the wound-healing assay, Uc.339 knockdown inhibited the invasion ability and vice versa, as indicated by the decrease in invaded cells (Fig. 2D, Fig. S1D).

### Uc.339 inhibited miR-339 expression by competitively binding to pri-miR-339

**Figure 3:**
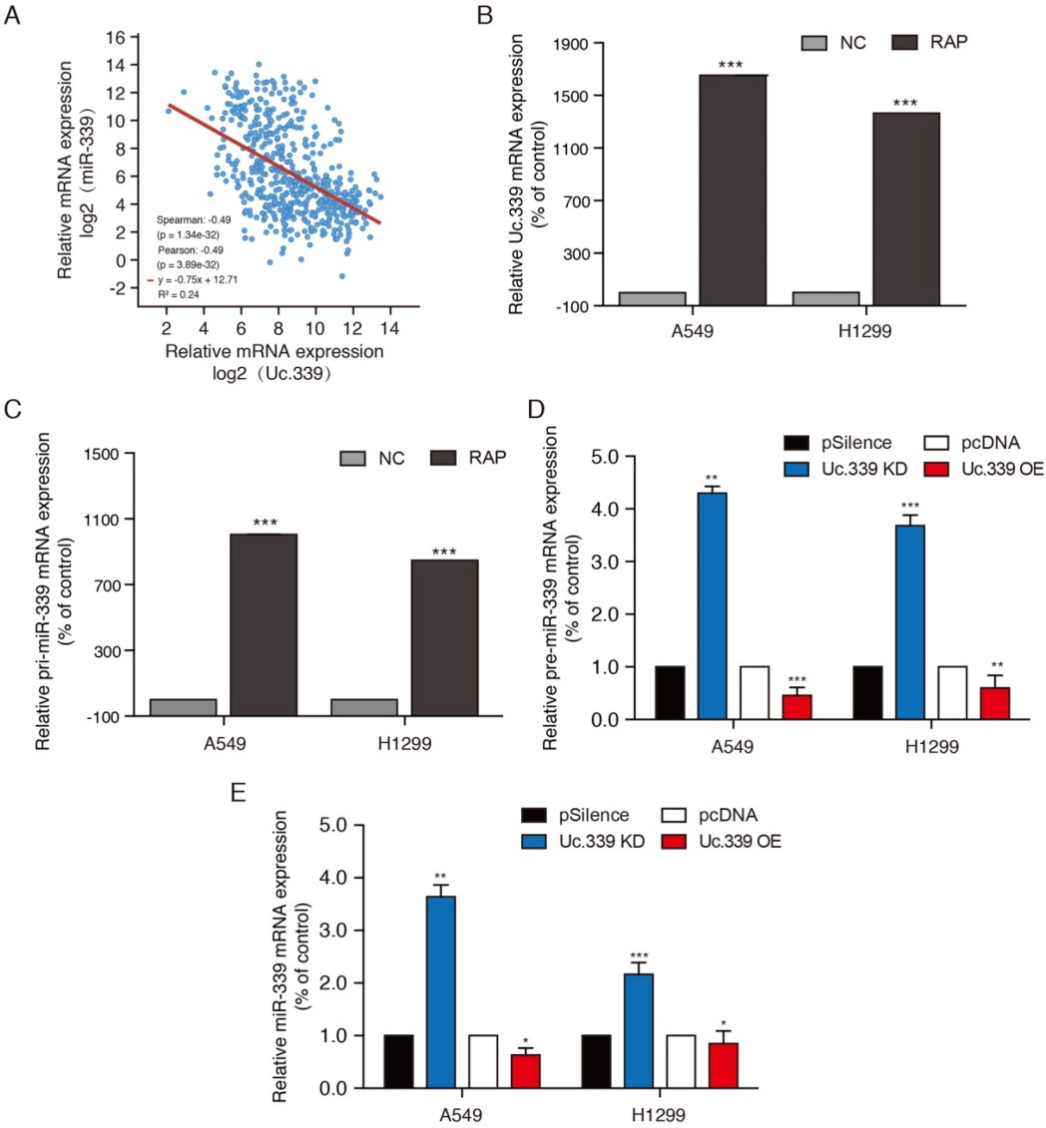
** Uc.339 inhibited miR-339 expression by competitively binding to pri-miR-339. a** Co-expression correlation analysis of Uc.339 and miR.339 in LUAD based on TCGA database. **b-c** RAP-PCR experiments were performed using the streptavidin probe group to immunoprecipitated in total cell extracts of A549 and H1299 cells, and relative enrichment was determined by real-time PCR. **d** qPCR analysis of pre-miR-339 in A549 and H1299 cells with Uc.339 shRNA or Uc.339 pcDNA3.1 (+). **e** qPCR analysis of miR-339 in A549 and H1299 cells with Uc.339 shRNA or Uc.339 pcDNA3.1 (+). Each bar represents the mean ± SD of three independent experiments; *P<0.05; **P<0.01; ***P<0.001.

To study how Uc.339 promotes the proliferation and metastasis of lung cancer, we aimed to explore the downstream target of Uc.339. In view of the fact that lncRNA T-UCR can complement the primary miRNA (pri-miRNA) through the hyper-conserved region, and regulate its shearing to reduce the expression of miRNA. Previous studies have found that the Uc.339 transcript sequence is complementary to miR-339. The level of miR-339 is negatively associated with a higher risk of lung cancer, which leads to miR-339 being considered a tumor suppressor gene. By comparing the mRNA expression of Uc.339 and miR-339 in lung adenocarcinoma cancer patients downloaded from the TCGA database, it was found that the expression of Uc.339 and miR-339 was negatively correlated (Fig. 3A). We hypothesized that Uc.339 may reduce the level of miR-339 through binding of pri-miR-339, thereby weakening the effect of miR-339 in inhibiting related carcinogenic targets. Therefore, we further explored the binding of Uc.339 and pri-miR-339 in A549 and H1299 cells. The results of RAP-PCR showed that Uc.339 can indeed bind to pri-miR-339 (Fig. 3B, 3C). Next, we knocked down or overexpressed Uc.339 in tumor cells by transfecting Uc.339 shRNA or Uc.339 pcDNA3.1 (+). The qRT-PCR results showed that when Uc.339 was knocked down, the expression of pre-miR-339 and miR-339 both increased apparently and vice versa (Fig. 3D, 3E). These results indicated that Uc.339 could competitively bind pri-miR-339 to prevent pri-miR-339 from being cut into pre-miR-339, thereby inhibiting the formation of mature miR-339.

### Uc.339/miR-339 targeted SLC7A11 and regulated its expression

**Figure 4:**
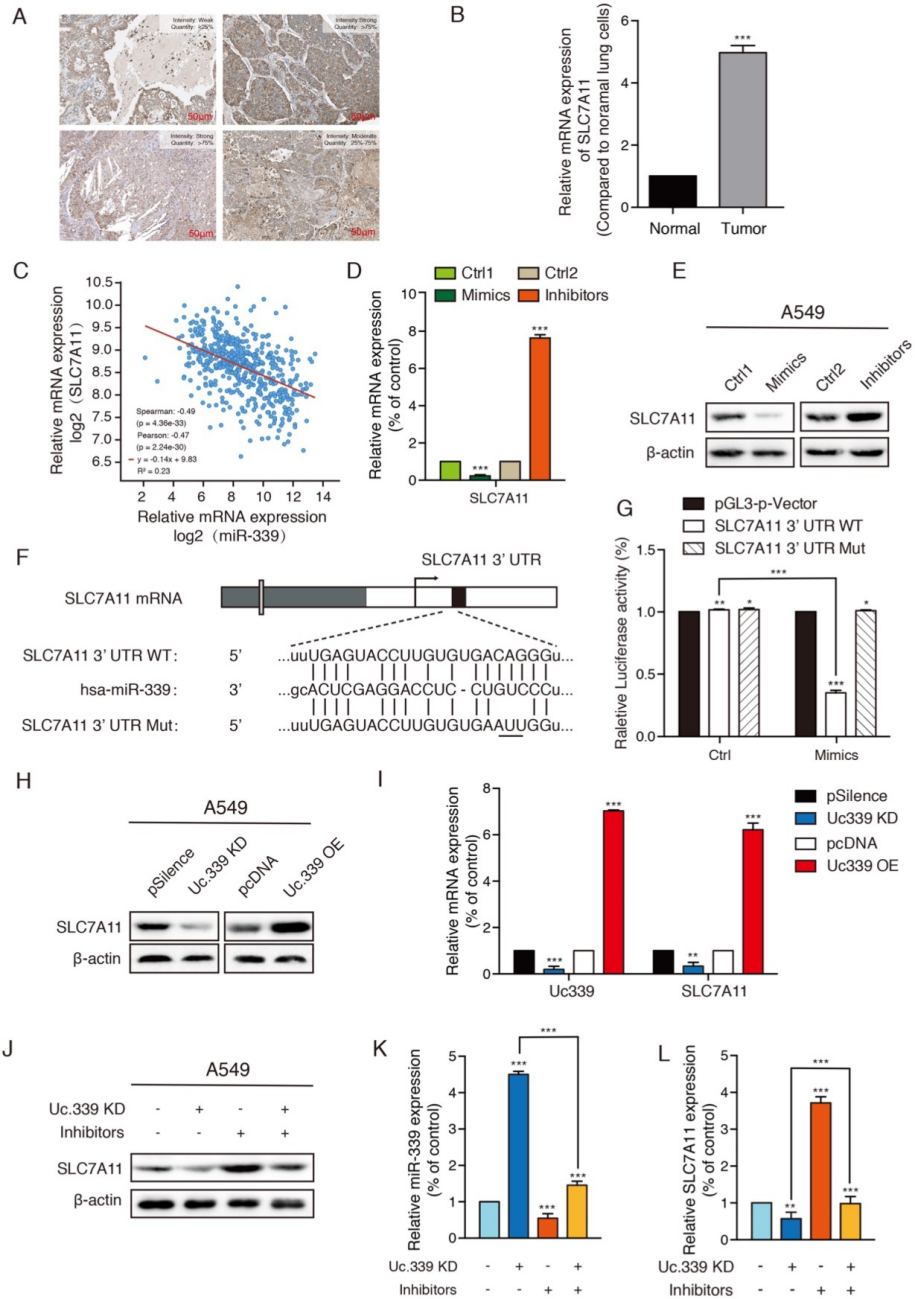
** Uc.339/miR-339 targeted SLC7A11 and regulated its expression. a** Expression levels of SLC7A11 protein in one healthy person and three patients with LUAD from the HPA database. Scale bar, 50 µm. **b** qRT-PCR analysis of SLC7A11 expression in cells extracted from healthy lung tissues and tumor tissues of mice. **c** Co-expression correlation analysis of miR.339 and SLC7A11 in LUAD based on TCGA database. **d-e** SLC7A11 protein and mRNA expression levels were found regulated directly by miR-339, as reflected by the decreased SLC7A11 expression in A549 cells after transient transfection of miR-339 mimics and increased SLC7A11 expression in A 549 cells after miR-339 inhibitors transfection. **f** The wild-type and mutant variant of the putative miR-339 target sequences of the SLC7A11 gene. **g** Two copies of the wild-type and mutant miR-339 target sequences were fused with a luciferase reporter and transfected into control oligonucleotide- and miR-339 mimics-infected A549 cells. miR-339 significantly suppressed the luciferase activity of the wild-type SLC7A11 3′ UTR. **h** Western Blot analysis of the indicated proteins from A549 cells treated with Uc.339 shRNA or Uc.339 pcDNA3.1 (+).** i** qRT-PCR analysis of SLC7A11 expression in A549 cells treated with Uc.339 KD plasmid or Uc.339 pcDNA3.1 (+). **j** Western Blot analysis of the indicated proteins from A549 cells treated with Uc.339 shRNA, with or without miR-339 inhibitors. **k-l** qRT-PCR analysis of miR-339 and SLC7A11 expression in A549 cells treated with Uc.339 KD plasmid, with or without miR-339 inhibitors. Each bar represents the mean ± SD of three independent experiments; *P<0.05; **P<0.01; ***P<0.001.

Compared with normal subjects, the expression of SLC7A11 in patients with lung adenocarcinoma is significantly increased. The high expression of SLC7A11 is accompanied by the high proliferation capacity and high invasion ability of lung cancer cells, which in turn is related to the cell ferroptosis involved in SLC7A11. The Human Protein Atlas (HPA) program revealed that SLC7A11 was positively detected via immunohistochemistry (IHC) staining in patients with LUAD (Fig. 4A). As showed in Fig. 4B, in the experimental lung metastatic model, the expression of SLC7A11 in cells extracted from tumor tissues was significantly increased compared with cells extracted from healthy tissues. We next extracted samples of 506 lung cancer patients in the TCGA database, and the analysis showed that the mRNA expression level of miR-339 in lung adenocarcinoma was highly negatively correlated with SLC7A11 (Fig. 4C). The results of Western Blot and qRT-PCR showed that there was a regulatory relationship between miR-339 and SCL7A11. Compared with the control group, the expression of SCL7A11 decreased in miR-339 mimics-transfected A549 and H1299 cells and increased in miR-339 inhibitors-transfected tumor cells (Fig. 4D, 4E; Fig. S2A, S2B). Then, we used the StarBase database to predict and analyze the combination of miR-339 and SLC7A11, and found they have strong binding ability. Therefore, we speculated that SLC7A11 is the downstream target of miR-339. To check whether miR-339 directly targets SLC7A11, the SCL7A11 3'UTR segment containing the miR-339 binding site was cloned to the luciferase reporting system. The plasmid lacking the miR-339 binding site was used as a negative control for luciferase activity (Fig. 4F). The reporter vector was transfected to the A549 and H1299 cells along with the miR-339 mimics. The results showed that miR-339 inhibited the luciferase activity in the construct with the SCL7A11 3'UTR segment containing the miR-339-binding site. No luciferase activity change was found when the cells were transfected with the negative control (Fig. 4G, Fig. S2C).

In the abovementioned experiments, we proved that Uc.339 inhibited the production of miR-339 by binding pri-miR-339, and miR-339 could directly target SLC7A11 and negatively regulate it. It's important to determine whether Uc.339 could regulate SLC7A11 through miR-339. Furthermore, we need to find out how the Uc.339/miR-339/SLC7A11 axis affects the metastasis of lung cancer. To confirm the function of Uc.339 in regulating SLC7A11, we first determined whether Uc.339 could adjust the expression of SLC7A11. We transfected Uc.339 shRNA and Uc.339 pcDNA3.1 (+) to achieve knockdown and overexpression of Uc.339 in A549 and H1299 cells. Both Western Blot and qRT-PCR analysis showed that the expression of SLC7A11 is positively regulated by Uc.339. When the level of Uc.339 in tumor cells was knocked down, the SLC7A11 expression also decreases and vice versa (Fig.4H, 4I; Fig. S2D, S2E). In order to further confirm that Uc.339 affects SLC7A11 through miR-339, we introduced miR-339 inhibitors. The results of Western Blot showed that miR-339 inhibitors could reverse the decrease in SLC7A11 expression caused by knockdown of Uc.339 (Fig. 4J; Fig. S2F). qRT-PCR also verified this conclusion (Fig. 4K, 4L; Fig. S2G, S2H). These results indicated that there was the Uc.339/miR-339/SLC7A11 axis, which mediated the repression of miR-339 and the subsequent activation of SLC7A11.

### Uc.339/miR-339/SLC7A11 axis mediated lung cancer metastasis by influencing ferroptosis *in vitro*

**Figure 5:**
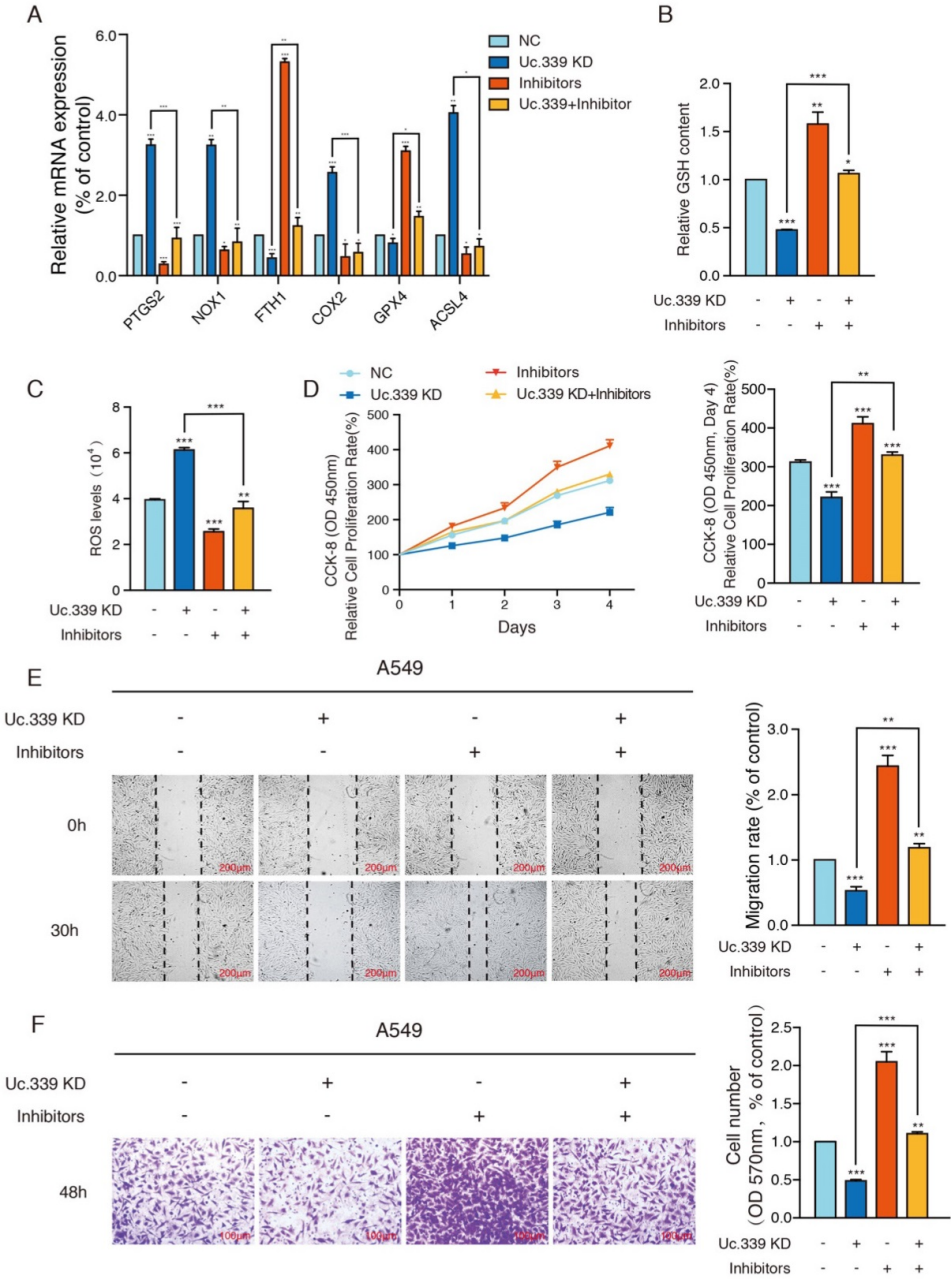
** Uc.339/miR-339/SLC7A11 axis mediated lung cancer metastasis by influencing ferroptosis *in vitro.* a** qRT-PCR analysis of PTGS2, NOX1, COX2, ACSL4, FTH1 and GPX4 in indicated tumor cells. **b** Relative GSH contents were determined and compared between the control and Uc.339 knockdown, with or without miR-339 inhibitors groups in A549 cells. **c** Relative ROS accumulation was determined with a multifunctional enzyme marker in A549 cells. d CCK-8 assay showing the proliferation rate of A549 cells with Uc.339 knockdown, with or without miR-339 inhibitors. **e** Wound-healing assay showing the migration ability of A549 with Uc.339 Knockdown, with or without miR-339 inhibitors. Scale bar, 200μm. **f** Transwell assay showing the invasion ability of A549 cells with Uc.339 Knockdown, with or without miR-339 inhibitors. Scale bar, 100μm. Each bar represents the mean ± SD of three independent experiments; *P<0.05; **P<0.01; ***P<0.001.

Previous studies have found that SLC7A11 is related to cell ferroptosis which affect the occurrence and development of tumors. Therefore, we first detected various ferroptosis-related genes to further understand the influence of Uc.339/miR-339/SLC7A11 axis on the development of lung cancer and its mechanism. qRT-PCR analysis showed that knockdown Uc.339, as well as decrease the level of SLC7A11, resulted in an increase in the expression of PTGS2, NOX1, COX2 and ACSL4, while the level of FTH1 and GPX4 decreased. The results indicated that Uc.339 knockdown promoted the ferroptosis of A549 and H1299 and this promotion is reversed by miR-339 inhibitors (Fig. 5A, Fig. S3A).

Ferroptosis is characterized by the accumulation of ROS, which is scavenged by GPX4 through conversion of reduced GSH into the oxidized form GSSG. Therefore, the expression of the GSH and ROS level were explored. GSH analysis found that compared with the control group, after knocking down Uc.339, the level of intracellular GSH decreased apparently, and this inhibitory effect disappeared after co-transfection of inhibitors (Fig. 5B, Fig. S3B). To calculate the accumulation of reactive oxygen species, a ROS kit was used to detect the level of ROS in tumor cells. Intracellular ROS levels were up-regulated in the Uc.339 KD group, and this promotion was also reversed after co-transfection of inhibitors (Fig. 5C, Fig. S3C). These experimental results all showed that the Uc.339/miR-339/SLC7A11 axis can affect cell ferroptosis.

To more intuitively explore the influence of Uc.339/miR-339/SLC7A11 axis on the metastasis of lung cancer, we used a CCK-8 kit to evaluate the level of cell proliferation. We observed that simply knocking down Uc.339 can effectively inhibit the proliferation of tumor cells, but this inhibition is reversed when miR-339 inhibitors are simultaneously transfected (Fig. 5D, Fig. S3D). Wound healing and Transwell analysis were performed on control cells and A549 and H1299cells with Uc.339 knockdown, with or without miR-339 inhibitors. In the wound-healing assay, the wound of Uc.339 knockdown cells were wider than that of the control cells, and the wound narrowed after being combined with the inhibitors (Fig. 5E, Fig. S3E). Similarly, Transwell analysis results show that Uc.339 can inhibit cell invasion, and the inhibitor can reverse this promotion (Fig. 5F, Fig. S3F). These results confirmed the function of Uc.339/miR-339/SLC7A11 axis in LUAD proliferation, migration and invasion *in vitro*.

### Uc339/miR-339/SLC7A11 axis influenced lung cancer metastasis *in vivo*

**Figure 6:**
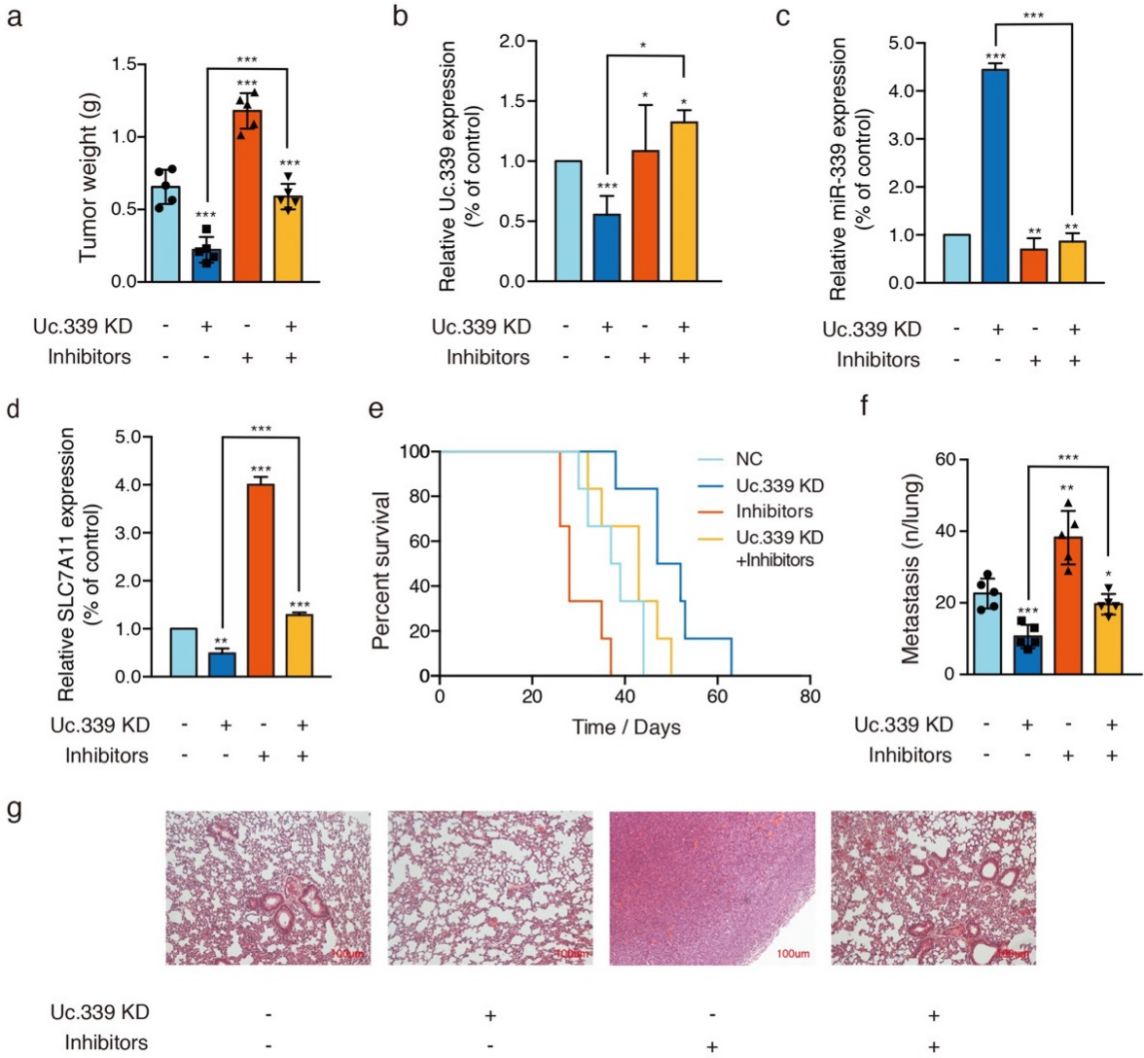
** Uc339/miR-339/SLC7A11 axis influenced lung cancer metastasis *in vivo***. **a** Relative tumor weights. **b-d** qPCR analysis of Uc.339, miR-339 and SLC7A11 expression *in vivo*. **e** Kaplan‐Meier survival curves for mice injected with Uc.339 knockdown, with or without miR-339 inhibitors LLC cells subcutaneously. **f** Pulmonary and liver metastasis was assessed after LLC cells injection through the lateral tail vein (n = 5 mice in each group). Metastasis was analyzed 28 days after injection of LLC tumor cells. Representative examples of the lungs (5 of each group) with metastatic foci were depicted. **g** Representative histologic evidence from tumor sections of the different groups. Four percent of paraformaldehyde-embedded lungs of all mice were cut completely, stained with hematoxylin and eosin, examined histologically and detected by microcopy. P values were determined by the log‐rank (Mantel‐Cox) test. Each bar represents the mean ± SD of three independent experiments; *P<0.05; **P<0.01; ***P<0.001.

The results of in vitro data suggested a potential role for Uc.339/miR-339/SLC7A11 axis in enhancing lung cancer cell metastasis. To analyze the relevance of the Uc.339/miR-339/SLC7A11 axis* in vivo*, we constructed two C57BL/6J mouse models. In the mouse xenograft model, we injected each group of cells into the inner thigh of the mouse by subcutaneous injection. Among them, the mice in group A were euthanized after 4 weeks. It was found that compared with the control group, the weight of the tumor formed by Uc.339 knockdown was markedly reduced; while the tumor weight formed by the single transfection inhibitor was higher than that of the control cells line; when the two were used in combination, effect of lung cancer cells by knocking down Uc.339 disappeared (Fig. 6A). The qRT-PCR results confirmed that the expression of Uc.339 and SLC7A11 decreased while the expression of miR-339 increased in the knockdown group, and the expression of SLC7A11 increased significantly after the miR-339 inhibitor was transfected (Fig. 6B-D). In group B, we adopted the method of natural death and recorded the survival curve of mice (Fig. 6E). It was found that the survival rate of mice after Uc.339 knockdown was higher than that of the control group. When the inhibitor was transfected, this pro-survival effect was inhibited.

Consistent with the results of in vitro experiments, in the experimental lung metastasis model of mice, as shown in Fig. 6F, compared with the control group, the number of lung metastases in mice injected with Uc.339 KD-LLC cells was significantly reduced. When the miR-339 inhibitor was transfected at the same time, this inhibitory effect on lung metastasis disappeared. The H&E staining results of lung tissue sections also verified this finding (Figure 6G). Taken together, these data suggested that UC.339 can promote lung cancer metastasis in vivo through the Uc.339/miR-339/SLC7A11 axis.

## Discussion

In this study, we identified a Uc.339/miR-339/SCL7A11axis which influences the lung metastasis caused by ferroptosis. We believe this is the first study clarifying the specific regulatory mechanism of Uc.339 and its downstream targets in lung adenocarcinoma.

Uc.339 is a transcribed ultra-conserved region lncRNA (lncRNA T-UCR). As a downstream response element of the classic tumor suppressor gene TP53, it can be silenced by TP53 in normal organisms. It is abnormally activated under pathological conditions, which promotes the occurrence and development of lung cancer. The study of Calin et al. found the carcinogenic effect of Uc.339 in HCC^11^; other studies also showed that Uc.339 was up-regulated in CRC^12^. However, its expression and function in lung cancer are still unclear.

In this study, we first analyzed the expression of Uc.339 and the overall survival of patients with lung adenocarcinoma based on the TCGA data obtained. We also detected the expression of Uc.339 in several lung adenocarcinoma cells (A549, H299 and PC-9) and primary cells extracted from lung tumor tissues of experimental lung metastases in mice. It was found that Uc.339 was up-regulated in patients and lung adenocarcinoma cells (Fig.1A-C) and the survival of patients with high Uc.339 expression was markedly shorter than that of patients with low Uc.339 expression (Fig.1D). We next conducted a series of *in vitro* experiments to detect the effect of Uc.339 on A549 and H1299 cells. Knockdown of Uc.339 inhibited the proliferation, migration and invasion of tumor cells and vice versa (Fig. 2A-D; Fig. S1A-D). Therefore, it represented an attractive target for lung cancer treatment. However, the effect of uc.339 on the metastasis of lung cancer and its specific regulatory mechanism are still unknown.

In view of our data indicating that Uc.339 behaves as a "growth metastasis promoting gene" in lung cancer, we next tried to determine the possible mechanism of this phenotype. The study of Ivan et al. found that T-UCR may act as a "bait" for complementary mature miRNAs. Subsequent studies also proved that transcribed uc.339 is upregulated in archival NSCLC samples, functioning as a decoy RNA for miR-339, -663b-3p, and -95-5p. miRNA has been considered an important transcriptional mediator in biological activities, including tumorigenesis and metastasis. Its synthesis and maturation are divided into two stages: miRNA gene is transcribed into pri-miRNA by RNA polymerase II, and it is cleaved by Drosha-DGCR8 cleavage complex into pre-miRNA of about 70 nucleotides in the nucleus. After the pre-miRNA is transported out of the nucleus by the nuclear transporter-Exportin5, it is cut into a mature miRNA of approximately 20 nucleotides in the cytoplasm by Dicer. To verify whether Uc.339 can inhibit the production of miR-339 by competitively binding pri-miR-339. First, we verified the correlation between Uc.339 and miR-339 expression in lung adenocarcinoma. Analysis of LUAD case samples in the TCGA database showed that Uc.339 is negatively correlated with miR-339 (Fig. 3A). To verify that Uc.339 can bind to pri-miR-339, we conducted RAP-PCR experiments, the results showed that Uc.339 can indeed bind directly to pri-miR-339 (Fig. 3B-C). When Uc.339 was knocked down, the expression of pre-miR-339 and miR 339 were significantly increased (Fig. 3D). These experimental results all confirmed that Uc.339 can inhibit the production of pre-miR-339 and miR-339 by competitively binding pri-miR-339.

Ferroptosis is a new form of nonautophagic and nonapoptotic programmed cell death in recent years^32^, ^33^. It can lead to a decrease in cell antioxidant capacity, accumulation of ROS and ultimately cause cell oxidative death, which are believed to be critical for development, homeostasis, disease occurrence, and treatment, such as malignant tumors^17^, ^32^, ^34^. The emerging role of ferroptosis linked cell metabolism and tumor suppression has been a topic of great interest^34^, ^35^. Many studies indicate that SLC7A11 acts as a key factor in modulating ferroptosis responses in human cancers. SLC7A11's influence on the occurrence and development of breast cancer, glioma, lymphoma and other cancers has also been reported in other researches^12^, ^36^-^38^. This suggests the correlation between SLC7A11 and cancer development. Emerging studies have found that the expression of its related gene SLC7A11 may be regulated by LncRNA. The results predicted by the StarBase database also showed that miR-339 may have a strong binding ability with SLC7A11 (Fig. 4F). We explored whether SLC7A11 was the direct target of miR-339 and the regulation of SLC7A11 by Uc.339/miR-339. It showed that SLC7A11 was up-regulated in patients or mice with lung adenocarcinoma (Fig. 4A-B) We detected the correlation between the expression of miR-339 and SLC7A11 from the perspective of mRNA and protein, and found that miR-339 negatively regulates the expression of SLC7A11 (Fig. 4C-E, Fig. S2A-B). Furthermore, our luciferase reporter assay determined that SLC7A11 is a direct target of miR-339 (Fig. 4G, Fig. S2C). It was showed that Uc.339 can positively regulate the expression of SLC7A11 through miR-339 by qRT-PCR and Western Blot (Fig. 4H-L, Fig. S2D-H).

To better understand the *in vitro* and *in vivo* significance of Uc.339/miR-339/SCL7A11 axis changes. We tested the expression of ferroptosis-related genes, GSH levels and ROS levels in Uc.339 knockdown A549 and H1299cells, with or without miR-339 inhibitors. Cell proliferation analysis, wound-healing assay and Transwell were also performed. The data all showed that knocking down Uc.339 can inhibit ferroptosis (Fig. 5A-C, Fig. S3A-C) and hinder the metastasis of tumor cells (Fig. 5D-F, Fig. S3D-F). After simultaneous transfection of miR-339 inhibitors, this tumor suppressor effect was inhibited. Consistent with the results of* in vitro* experiments, in the mouse xenograft model, compared with the control group, the mice in the Uc.339 KD group had smaller solid tumors (Fig. 6A), indicating that knocking down Uc.339 could inhibit tumor formation. But after transfection of miR-339 inhibitors, this inhibitory effect disappeared. The mouse survival curve also proved this conclusion (Fig. 6E). We then tested the contents of Uc.339, miR-339 and SCL7A11 in solid tumors, and confirmed the regulatory relationship between them in vivo (Fig. 6B-D). The experimental results of the mouse lung metastasis model also verified the previous conclusions (Fig. 6F-G).

Our research showed that the increased expression of Uc.339 prevented the Drosha-DGCR8 splicing complex from binding to pri-miR-339, thereby inhibiting the production of mature miR-339. The imbalance of miR-339 led to the up-regulation of the ferroptosis-related gene SLC7A11 and the reduction of ferroptosis levels, which promotes the metastasis of lung adenocarcinoma.

Previous studies have found that the high expression of LncRNA T-UCR Uc.339 could promote tumor metastasis, and found that there was a regulatory relationship between LncRNA T-UCR Uc.339 and miR-339^39^. However, we have further explored LncRNA T- UCR Uc.339 regulates the specific mechanism of miR-339, that is, the expression of Uc.339 increases during the metastasis of lung adenocarcinoma. It could competitively bind with pri-miR-339 to inhibit the cleavage of pri-miR-339 into mature miR-339, thereby inhibiting the expression and function of miR-339 to further drive lung cancer metastasis. At the same time, it is the first to clarify the reason for the negative correlation between miR-339 and SLC7A11 expression in lung cancer, and for the first verification that the inhibition of miR-339 led to increased expression of SLC7A11 and weakens ferroptosis, which constituted an important carcinogenesis mechanism for lung adenocarcinoma metastasis. In the field of lung cancer, the regulatory axis of LncRNA T-UCR Uc.339/miR-339/SLC7A11 was discovered for the first time, and its regulatory effects and mechanisms on lung adenocarcinoma metastasis were explored *in vivo* and* in vitro*. All in all, our research results revealed the Uc.339/miR-339/SCL7A11 axis that mediates the ferroptosis of lung cancer cells, and clarified the specific mechanism of Uc.339 that promotes the metastasis of lung cancer. The Uc.339/miR-339/SCL7A11 axis may become an important indicator for early diagnosis, screening and progress monitoring in clinical practice, and the key factors may become the new target for the prevention and treatment of lung adenocarcinoma.

## Supplementary Material

Supplementary figures.
