# Supplementary material for: LncRNA T-UCR Uc.339/miR-339/SLC7A11 Axis Regulates the Metastasis of Ferroptosis-Induced Lung Adenocarcinoma
Source: J Cancer. 2022 Mar 28;13(6):1945–57. doi: 10.7150/jca.65017 (PMC8990432; doi:10.7150/jca.65017)
Supplement: Supplementary file 1 — Supplementary figures. [file jcav13p1945s1.pdf]

Supplementary file：

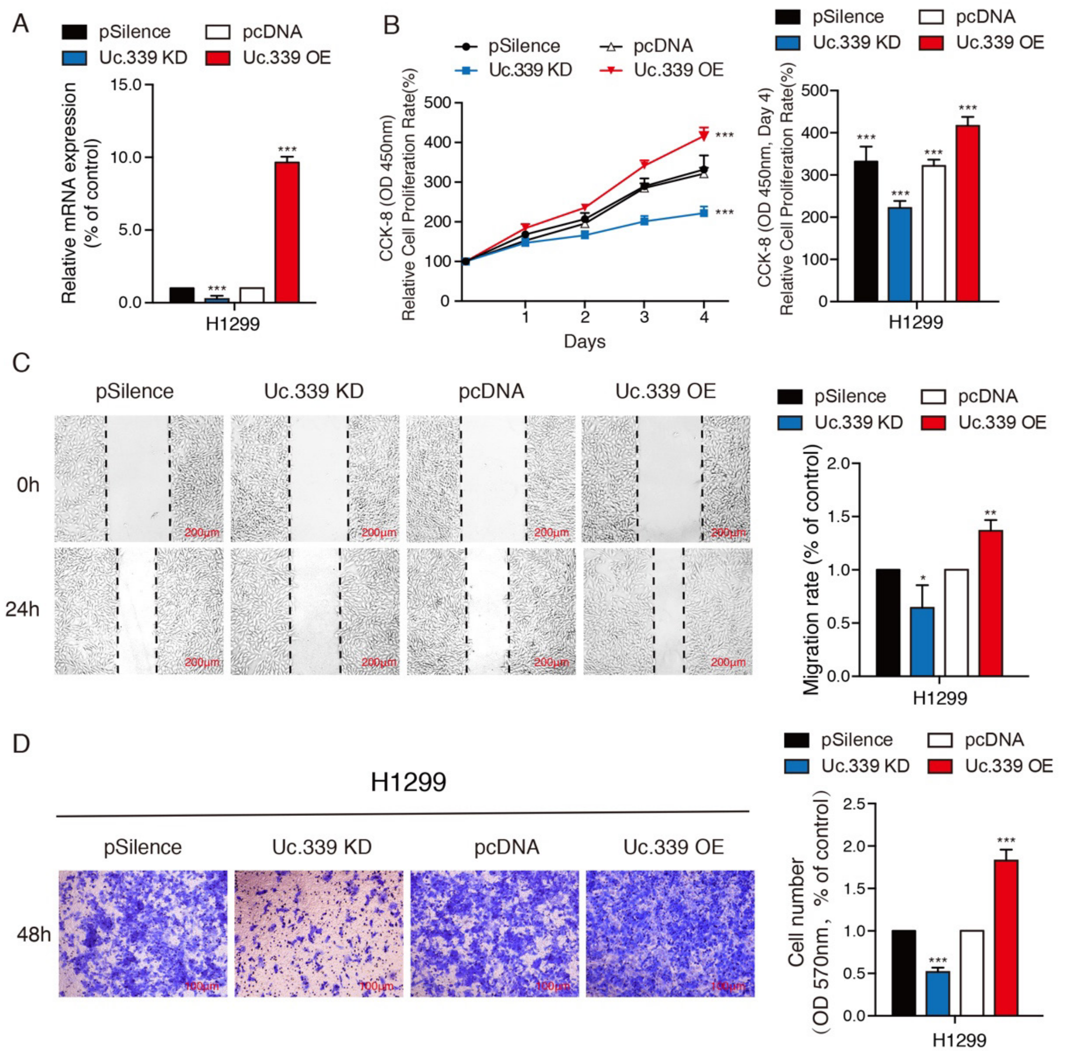

Supplementary Figure 1: Uc.339 promoted the proliferation and metastasis of lung adenocarcinoma cells

a qRT-PCR analysis of Uc.339 expression in H1299cells with Uc.339 knockdown or Overexpression. **b** CCK-8 assay showing the proliferation rate of H1299 cells with Uc.339 knockdown or overexpression. **c** Wound-healing assay showing the migration ability of H1299 with Uc.339 Knockdown or overexpression. Scale bar, 200μm. **d** Transwell assay showing the invasion ability of H1299 cells with Uc.339 Knockdown or overexpression. Scale bar, 100μm. Each bar represents the mean ± SD of three independent experiments; **P*<0.05; ***P*<0.01; ****P*<0.001.

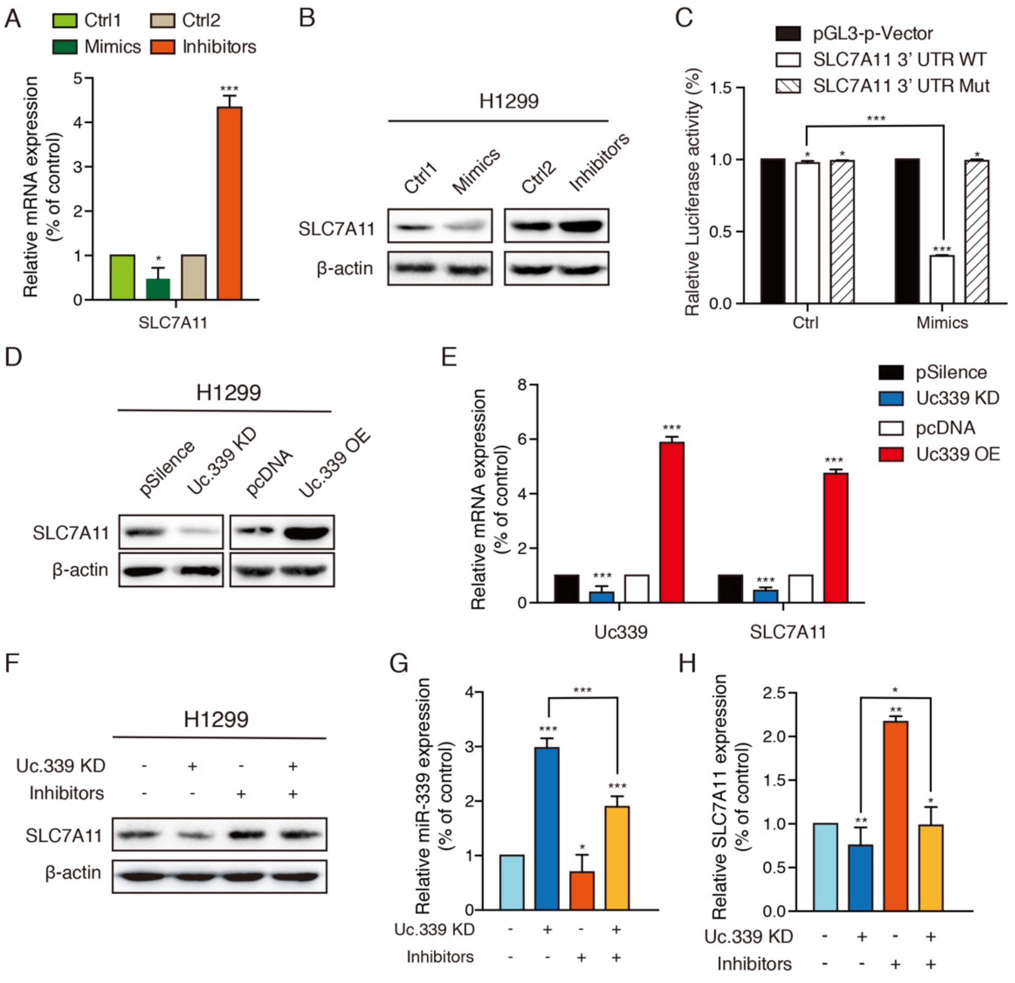

Supplementary Figure 2: Uc.339/miR-339 targeted SLC7A11 and regulated its expression

a-b SLC7A11 protein and mRNA expression levels were found regulated directly by miR-339, as reflected by the decreased SLC7A11 expression in H1299 cells after transient transfection of miR-339 mimics and increased SLC7A11 expression in H1299 cells after miR-339 inhibitors transfection. **c** Two copies of the wild-type and mutant miR-339 target sequences were fused with a luciferase reporter and transfected into control oligonucleotide- and miR-339 mimics-infected H1299 cells. miR-339 significantly suppressed the luciferase activity of the wild-type SLC7A11 3′ UTR. **d** Western Blot analysis of the indicated proteins from H1299 cells treated with Uc.339 shRNA or Uc.339 pcDNA3.1 (+). **e** qRT-PCR analysis of SLC7A11 expression in H1299 cells treated with Uc.339 KD plasmid or Uc.339 pcDNA3.1 (+). **f** Western Blot analysis of the indicated proteins from H1299 cells treated with Uc.339 shRNA, with or without miR-339 inhibitors. **g-h** qRT-PCR analysis of miR-339 and SLC7A11 expression in H1299 cells treated with Uc.339 KD plasmid,

with or without miR-339 inhibitors. Each bar represents the mean ± SD of three independent experiments; *P<0.05; **P<0.01; ***P<0.001.

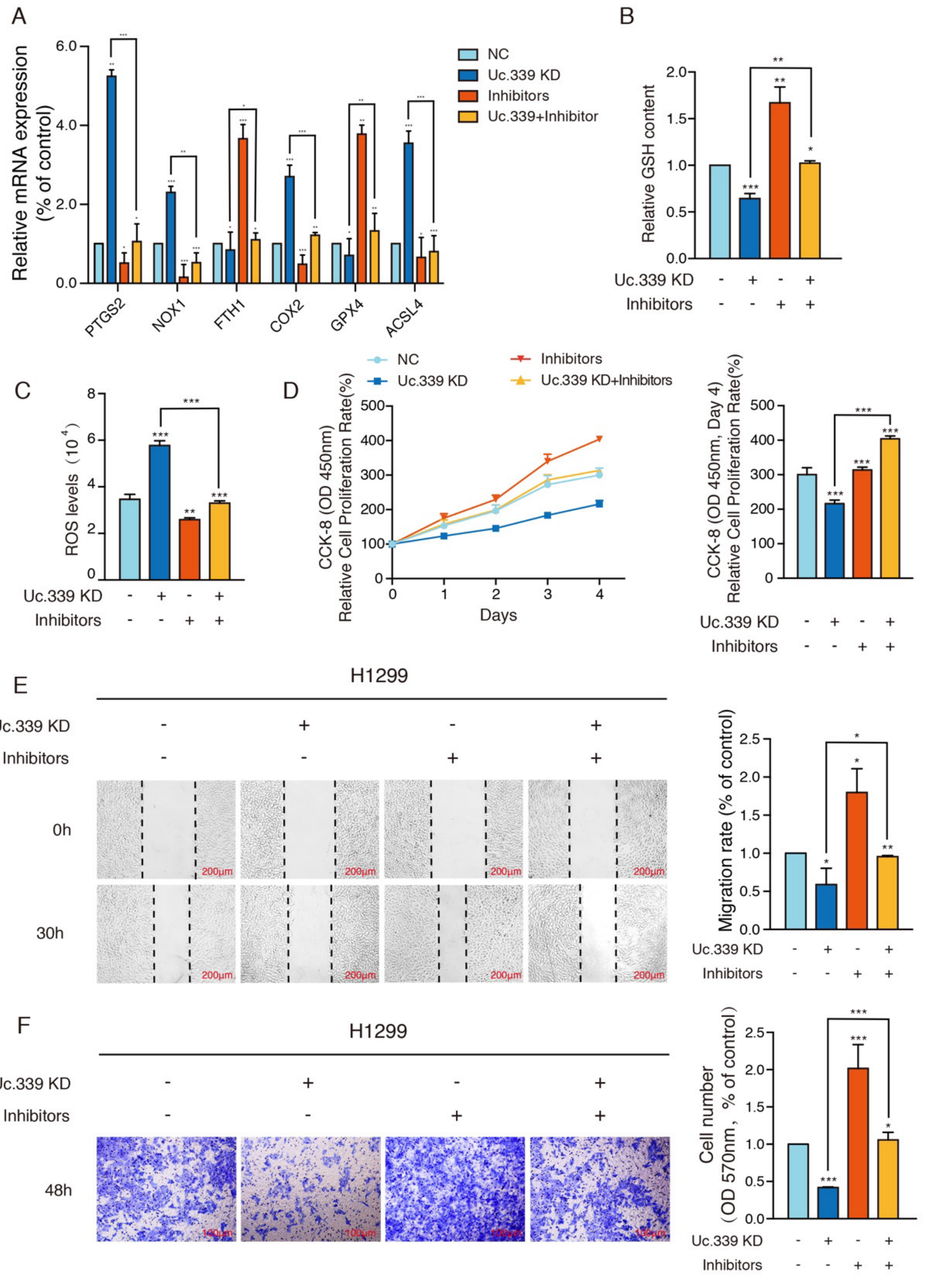

Supplementary Figure 3: Uc.339/miR-339/SLC7A11 axis mediated lung cancer metastasis by influencing ferroptosis *in vitro*

a qRT-PCR analysis of PTGS2, NOX1, COX2, ACSL4, FTH1 and GPX4 in indicated tumor cells. **b** Relative GSH contents were determined and compared between the

control and Uc.339 knockdown, with or without miR-339 inhibitors groups in H1299 cells. **c** Relative ROS accumulation was determined with a multifunctional enzyme marker in H1299 cells. d CCK-8 assay showing the proliferation rate of H1299 cells with Uc.339 knockdown, with or without miR-339 inhibitors. **e** Wound-healing assay showing the migration ability of H1299 with Uc.339 Knockdown, with or without miR-339 inhibitors. Scale bar, 200μm. **f** Transwell assay showing the invasion ability of H1299 cells with Uc.339 Knockdown, with or without miR-339 inhibitors. Scale bar, 100μm. Each bar represents the mean ± SD of three independent experiments; **P*<0.05; ***P*<0.01; ****P*<0.001.
